# Reports of Cases Occurring in the Medical Ward of the Buffalo Hospital

**Published:** 1857-05

**Authors:** A. W. Nichols

**Affiliations:** Assistant in the Department of Clinical Medicine


### BUFFALO MEDICAL JOURNAL

AND

### MONTHLY REVIEW.

VOL. 12. MAY, 1857. NO. 12.

### ORIGINAL COMMUNICATIONS.

ART. I.—*Reports of Cases occurring in the Male Medical Ward of the Buffalo Hospital of the Sisters of Charity, during the Session of the Medical College for the Winter of* 1856–7. AUSTIN FLINT, M. D., *Prof. of Clinical Medicine and Pathology, Attending Physician.* Reported by the Assistant in the Department of Clinical Medicine, A.W. NICHOLS, M. D.

No. II. *Albuminuria: Bright's Disease: Desquamative Nephritis (acute and chronic) of Johnson.*

The term *albuminuria*, denotes merely the presence of a constituent of the blood in the urinary secretion. It does not exist in healthy urine, consequently it becomes a sign of disease pointing to a morbid condition of the secreting organ—the kidney. Dr. Bright, in 1837, first described the serious organic lesion of the kidney, with which albuminous urine is so frequently—*but not always*—associated; and since that time, the morbid condition inferred to exist from the presence of albumen in the urine, has been called Morbus Brightii. But it has since been discovered that albumen may be present in the urine and yet no organic lesion of the kidney exist. Still later, Dr. Johnson has called this diseased condition, acute or chronic *Desquamative Nephritis*, from the microscopical appearances which the urine presents, and from the changes which the kidneys undergo. The three following cases are interesting in connection with the more recent investigations aud with the more rational pathological views respecting this disease. To avoid tedium, all circumstances having no bearing on the subjects under discussion, will be omitted: and to bring before the reader the signs or symptoms attendant on the morbid condition commonly known as Bright's Disease, they will be arranged analytically, according to the various organs or tissues in which they were presented to notice.

1. *Previous condition of Patients.*

*a.* Case I, had a pulmonary affection about a year ago; Case II, had two distinct attacks of intermittent fever, the last about six months since; Case III had an attack of sub-acute rheumatism about five months since. But all three of the patients returned to their work, and enjoyed tolerable health, up to a short time previous to admission to hospital.

*b.* Case I, farmer, aged 36 years; Cases II and III, laborers, aged respectively, 24 and 26 years.

*c.* All had been in the habit of indulging more or less in alcoholic liquors.

2. *Symptoms prior to abandonment of occupation.*

*a. Pain.* Cases I and III had sharp and shooting pains in lumbar region for several weeks, at least.

*b. Epistaxis.* All three cases presented this symptom; most frequent and severe in No. 3; noticed first about six weeks before giving up work, in Nos. 1 and 3.

*c. Cephalalgia* present in the three cases; dull, frontal, and most severe at night-time; most marked in No. 3. This was the symptom on account of which all three of the patients took to bed.

3. *Symptoms referable to the Digestive System.*

*a. Appetite.* Anorexia in all the cases; thirst moderate, in Nos. 1 and 2.

*b. Nausea and vomiting,* both present in Nos. 2 and 3, and usually in the morning, at night, or after meals.

*c. Diarrhœa.* Occasionally in Nos. 1 and 3.

4. *Symptoms referable to the Circulatory System.*

*a. Heart.* In all three cases, no bellows-murmur was noticed; the sounds of heart were pure; in Case III, pericarditis was developed while in hospital. (*Vide Remarks.*)

*b. Blood-vessels.* Pulse varied from 60 to 88; not well developed in No. 1.

*c. Capillaries.* Capillary congestion of face, present in No. 1.

5. *Symptoms referable to the Respiratory Organs.*

The respirations were labored and more frequent in Nos. 1 and 2, in whom dropsy was present.

6. *Symptoms referable to the Nervous System.*

*a. Cephalalgia* continued in all three cases after coming to the hospital; was most severe at night, chiefly in the forehead, and dull in character; it was most marked in No. 3.

*b. Sleep.* There was restlessness or want of sleep in each of the three cases, at first.

*c. Vision.* In Nos. 1 and 3 there was partial amanrosis, most marked and persistent in No. 3.

*d. State of Mind.* No delirium manifested; mind clear; No. 3 was inclined to drowsiness, soon after his admission, and this increased up to supervention of pericarditis. (*Vide remarks.*)

7. *Symptoms referable to the Skin.*

Skin was warm and mellow, in Cases II and III; warm and dry, and no perspiration for some weeks, in Case III; skin in Nos. 2 and 3 was pallid or sallow; prolabia in No. 3, pallid; in No. 1, a dusky hue to skin of face.

8. *Symptoms referable to the Urinary Organs.*

*Urine.* Generally increased in Nos. 1 and 3; color natural, in Nos. 1 and 3; high colored in No. 2; sediment not much in Nos. 1 and 3; considerable in No. 2. Albumen: urine highly albuminous in all the cases. (*Vide remarks.*)

*Microscopic appearances of Urine.*

Case I: no blood corpuscles seen; *Epithelial casts* of tubuli uriniferi visible, but not numerous.

Case II: no casts visible, but an abundance of blood corpuscles.

Case III: urine not examined under microscope.

9. *Symptoms referable to no particular organ.*

*Dropsical Effusions.* In No. 1, anasarca appeared on backs of hands, only ten days prior to admission; it was noticed on the hands, feet and face, for two days previous to appearance of ascites; when admitted to hospital, there was slight puffiness of face, slight infiltration of ocular conjunctiva of both eyes; moderate anasarca of upper, and considerable of lower, extremity; slight ædema over sternum; small effusion in pleuræ and some in abdomen.

In No. II, four weeks prior to admisson noticed slight swelling of both feet, then of legs, next of thighs, then of penis and scrotum; after this, œdema of hands, and considerable of face; when admitted to hospital, moderate œdema of hands, considerable of face, slight over sternum and sides of chest, moderate degree of ascites, inferior extremities and penis and scrotum very much enlarged by effusion.

In No. III, about four weeks prior to admission, noticed slight swelling of feet, then moderate swelling of face, next of hands; when admitted, there was no evidence of anasarca anywhere, nor of any serous effusion in chest or abdomen. (*Vide remarks.*)

*Febrile movement.* Chilliness with rigors, and followed by slight febrile movement, was noted only in No. 3, and occurring prior to admission.

*Remarks.* The phenomena which appear in order of succession, may be different in different cases, according to the acute or chronic nature of the disease.

That condition of the kidney called Bright's kidney or Bright's disease, has been found to present different appearances both to the naked eye and when examined under the microscope. In that form of the disease which has been considered as *acute*, the kidneys appear more vascular, not generally so, but in patches; the "surface is smooth and the capsule readily peels off;" in some places, the capsular surface is pale; in other spots, the vessels are filled with blood, producing a scarlet or slate color. On cutting into the kidney, the cortical and medullary portion appear distinct: the former presents much the same appearance as is seen on the capsular surface, "congestion and anæmia." The kidney may be "firmer than natural." The medullary cones appear congested; their bodies seem compressed, "while the bases of the cones are spread out into the cortical substance, having the form of a *sheaf of wheat;*" the cortical portion appears narrowed or compressed, likewise.

In the *chronic* form, the appearances will vary according to the duration of the disease. Generally, some time elapses before an examination of the kidney is afforded. The kidney then is diminished in weight and bulk, affecting principally the cortical substance, so that on section the medullary portion approaches the capsular surface much nearer than normally is the case. The surface of the kidney loses its smoothness and becomes *granular.*

In those instances where albumen is found in the urine and accompanying or following some morbid condition of the system as scarlatina or some cases of pregnancy, the presence of albumen does *not necessarily* denote a diseased state of the kidney sufficient to constitute what is commonly called Bright's disease. The albuminuria in such cases, may be merely an evidence of simple congestion of that organ or of an anæmic condition of the system generally. But, wherever albumen exists for considerable time and in great abundance in the urine, then there is good ground for suspecting organic lesion of the kidneys.

The patient may suffer from an acute attack.* The disease may be chronic, as was the case probably in all of these patients.

In the chronic form of the disease, the symptoms may vary according as the function of the kidneys has been more or less compromised by the morbid condition.

Usually, there is presented the following order of symptoms: pain in the loins, severe and lancinating at times; dull pain, most severe at night, and in the forehead; loss of strength; epistaxis; impairment of vision, as partial amaurosis, or muscæ volitantes; noise in the ears or slight deafness; skin dry and warm, or deficient in perspiration; pallor of the face and lips, or sallowness of the skin; capillary congestion of the hands and face, diffused

* None of these cases appear to have been in the acute stage of the disease at the time of admission to hospital.

and dusky in color; more or less thirst; anorexia commonly; nausea and vomiting at times, generally in the morning, at night, or after a meal; diarrhœa occasionally; a disposition to sleep or drowsiness; restlessness at night; slight chills or rigors, followed by febrile movement; the urine diminished in quantity at first, and then increased, or not differing from that of health in quantity or color; there may be cough and expectoration of transparent mucus; the appearance of anasarca in the *hands or feet at first*, then in legs, face, chest, and abdomen; then serous effusions as in the pleura, peritoneum, pericardium, and arachnoid membrane; the face exhibits more or less puffiness, especially about the eyelids, and most on the side the patient lies upon; infiltration more or less of the ocular conjunctiva, producing varying degrees of chemosis; more or less pitting on pressure over sternum; enlargement of penis and scrotum; a greater or less amount of pleural effusion or of ascites, recognized by percussion: or there may be not much, if any, appearance of dropsy; but the sudden development of serous inflammation; as in Case, No. III, where *pericarditis came on suddenly*; and during the attack of pericarditis, the appearance of *rheumatism*, transient and limited to a single joint. In Case, No. III, the drowsiness or disposition to sleep continued, the patient falling asleep in his chair, his mind seeming dull or heavy; and whilst the pericarditis was proceeding to a favorable termination, the sudden supervention of *coma*, no convulsions manifested, and death taking place very soon after its appearance. Such seemed to be the course of the disease in the three cases up to the period of the development of the pericarditis in the last case.

The two first cases were treated with the potassæ bitartras, diluted in a large quantity of water, and employed as a drink during the day, there being considerable anasarca and some serous effusion present. The kidneys responded readily to this remedy, and the dropsical collections entirely disappeared. The acidum gallicum was then commenced in doses varying from gr. x to ℈ i, three times daily: with a generous diet. In No. I the gallic acid was continued ten days, An examination of the urine under the microscope, furnished no evidence of blood corpuscles nor of the previously observed epithelial casts; the urine became clear, almost like spring water. This patient left the hospital just one month after his admission, feeling sufficiently strong to engage in daily work, having an aspect of health, and there being no evidence of any dropsical collection in the cellular tissue or serous membranes. He subsequently was not heard from.

In No. II, after the cream of tartar had been continued until the anasarca and ascites had entirely disappeared, for about ten days time, the gallic acid was employed for nearly three weeks, when anasarca reäppearing, the potassa was resumed with the effect of again dispelling the effusion; under the use of this remedy, the patient passed daily a large quantity of water; and improving in strength and having no return of dropsy, in two weeks he left the hospital to resume his business. Subsequently he returned, and is now under treatment for the same disease.

What was the effect on the urine during the employment of the potassa? The quantity was considerably increased; the amount of albumen in the secretion for twenty-four hours, was not affected. What change occurred in the urine in each of these cases, when the gallic acid was substituted for the cream of tartar? In Case I, after this agent had been employed for ten days, in the dose of one scruple, three times daily, only a *faint trace of albumen* was discoverable. In Case II, the amount of albumen was not appreciably diminished, but remained about the same: this still continues to be the case. It was given in doses of ten grains, three times daily, in this case, and was suspended on account of the reäppearance of anasarca. It would be interesting to inquire into the effect of gallic acid in the above case; of course it was absorbed and carried into the circulation, and then, probably, during the process of secretion in the kidney, exerted its beneficial effect as an astringent, thereby preventing the farther outpouring of albumen; in order to produce this result, it must have been combined with some material formed in or contained in the blood, capable of changing it into tannic acid; for when gallic acid is applied externally, it is not, like tannic acid, astringent.*

The *third case* was not examined in the foregoing analysis, farther than to the period of the sudden development of a serous inflammation, pericarditis. From this point the case furnished, in its course, a variety of highly interesting phenomena; and, after decease, disclosed appearances, for a correct appreciation of which, recourse must be had to the pathology of the disease. An enumeration of the order of phenomena presenting themselves, in *Bright's disease*, has been attempted already in this article. Those which occurred in this case, after the supervention of pericarditis, are there adverted to. At this point, however, greater detail is necestary. Case, No. III, presented nothing unusual in the previous history or condition on admission to hospital, demanding a separate consideration from the other cases. He had had not much general dropsy: there was partial amanrosis; vomiting was present; considerable epistaxis existed; but he appeared drowsy or

* For farther observations on the supposed effect of this agent, see "Headland on the Action of Medicines." Philadelphia. 1853.

inclined to sleep. On the night of the fourth day after admission, (having retired as well as he had been since his admission,) he was attacked with pain in the precordia, shortness of breathing, and increased pain in the head. At the morning visit, *ten hours after the sudden attack of pain and shortness of breathing*, physical exploration afforded the *evidence of pericarditis*, with the *friction-sound* most beautifully exemplified, there being as yet not much effusion. The extent to which the dullness on percussion, (over precordia), and the existence of the friction-sound, were manifested, together with the absence of respiratory sound, were delineated on the chest by Prof. Flint. But, associated with this interesting phenomena, was another no less interesting. During the morning, before the usual visit, the left wrist became painful, tender to the touch, red, and swollen; in fact, *acute articular rheumatism* was established; this secondary disease, so called, was transient, and had disappeared entirely in two or three days: it was confined exclusively to the above-named joint. The patient continued to improve for about three days after the appearance of the pericarditis; this disease was progressing to a favorable termination; not much effusion had taken place; the friction-sound was somewhat more intense and diffused than it had been. It should be observed, that the *sounds of the heart*, before and after the appearance of the pericarditis, were *pure*; there was no evidence of valvular lesion. But, notwithstanding this condition of the patient, he continued to be drowsy, and his mind seemed to be dull. On the evening of the fourth day after the attack of the pericarditis, singultus and dysphagia appeared, and increased so that the attempt to swallow seemed to occasion convulsive efforts; the respiration became stertorous; he fell into a comatose condition, and died in fifteen hours after the appearance of the dysphagia.

The *autopsy* being confined to the chest and abdomen, was made about nine hours after decease.

Portions of the notes of the autopsy, made in the hospital record, by Prof. Flint, relating to the heart and to the kidneys, are transcribed, as follows:*

"On removing the sternum, the heart and pericardial sac were found to occupy a space, (*without* any intervening lung,) *precisely corresponding* to that delineated on the chest during life, the boundary lines, in ink, remaining on the cadaver." There was some effusion of serum into both pleural sacs, more in the left than in the right, but no evidence of pleuritis. The pericardial sac contained some liquid, but there was none anterior to the

* Much of these reports are transcribed from the record kept at the hospital by Prof. F. and his assistant. N.

heart in situ; on opening it, the "evidence of recent pericarditis was finely manifested." There were successive layers of fibrin, reticulated or like lace-work, over the greater portion of the visceral surface which was roughened; in other portions, as at the base, it was in a membranous layer, and at others rolled up into bands; the fibrin was recent, not adherent, not firm nor organized; the parietal surface was likewise roughened.

"The heart was considerably enlarged: the *enlargement was by hypertrophy.* the cavities did not appear much, if at all, increased in size."

The measurements and weight were as follows:

"Weight, after removal of clots, 24 oz; length, from point where aorta appears to apex, 6 inches; width, at widest part, $6\frac{3}{4}$ in.; thickness of left ventricle, at thickest point, $1\frac{1}{8}$ in; do. of right ventricle, $\frac{3}{8}$ in. The mitral and aortic valves were healthy.

"The kidneys presented a fine illustration of granular degeneration. They were evidently under size: each weighed alike, four ounces; they were firm and resisting. The capsule was detached without difficulty, no portion of the organ remaining attached. The surface was everywhere uniformly granular, not merely granular deposit distinguished by difference of color, but * * * * * the exterior surface thickly studded with granular projections of about the size of a large pin's head. Both kidneys presented as nearly as possible the same appearance. Internally, the cortical portion was evidently diminished. The distance from the expanded extremity of the pyramids to the exterior surface, was small; but a measurement was not made. The expanded extremities of the pyramids presented the *frayed appearance,* compared by Rokitansky to a *sheaf of wheat.* The deposit did not appear to have invaded to much extent the pyramids or the spaces between them."

Now what were the causes of the pericardial inflammation, of the hypertrophy without dilatation or valvular lesion, of the rheumatic attack, and of the coma? The answer or answers to this question, involve an inquiry into the nature of the disease. That the degeneration of the kidney, is not the first link in the chain of morbid conditions, is generally admitted. The disease exists prior to this. Is it an inflammation of the kidneys, more or less acute or chronic in form? This is the views taken by Dr. Johnson in a valuable treatise published in London, in 1852. He calls this disease "Desquamative nephritis, acute or chronic." The three cases before the reader, would come under the head of "chronic desquamative nephitis," according to him. As the views of Dr. Johnson are rational and drawn from actual study of the disease, in all its details, some general idea of them may be presented at this time; leaving the reader to consult his work, the result of much study and of patient investigation. He "adopts as a central truth, that all the changes of structure commence in the secreting cells of the gland, and are the result of an effort made by the cells to eliminate from the blood some abnormal products—some materials which do not naturally enter into the composition of the renal secretion."

Now, as to the nature of this material, in many cases. he adds, "we know nothing certain," "and the evidence that there is some *materies morbi* which excites the renal disease is derived: 1st, from a consideration of the circumstances under which the disease occurs;" as he supposes, that "all the causes of renal disease have this common feature, that they tend to produce a morbid condition of the blood." 2d, "from analogy"; and 3d, it might be added, that this being one of the so called symmetrical diseases, (or those diseases which affect, at the same time, the same organs or parts on both sides of the body,) it would be fair to presume that the proximate cause of the disease was a morbid condition of the blood, as is the case in articular rheumatism, and pulmonary tuberculosis. He believes, that "the cells in striving to separate the strange materials, become modified in their action and nutrition, and being rapidly thrown off into the tubes, are thence removed by the current of liquid, and appear in an *entire form in the urine*." The process of secretion, in the mean time, becomes checked, and with this "impeded secretion, there is, as a necessary consequence, *retarded circulation*." The process of secretion being compromitted, the blood becomes loaded with excrementitious matter, and the "Malpighian capillaries and the arteries become distended," from the impeded capillary circulation.* The liquor sanguinis now escapes from the engorged Malpighian tufts, rendering the urine albuminous, a transudation of fibrin occurs "which coagulates in the tubes, entangling on its surface some of the desquamated epithelial cells," forming the *epithelial casts;* some of the capillaries give way, and then the blood escaping, furnishes the corpuscles which are seen in the urine. If the disease progresses and becomes chronic, the process of inflammation continuing, at length, the epithelial cells become disintegrated, and a granular material is discovered in the urine, in the form of casts; this granular substance being the remains of broken down epithelial-cells which surround

* At one of the clinical lectures on this subject, Prof. F. called attention to the fact that in Case, No. III, whilst there was an increase in the amount of albumen in the urine, the amaurosis diminished; this going to support the "hypothesis that the amaurotic trouble *was not* due to the *anæmia*, but to *retained urinary products*."

the fibrinous casts. Still farther advanced, casts of a "waxy appearance," are seen in the urine, denoting that some of the urinary tubules have been entirely deprived of their epithelial covering and rendered useless in the process of secretion. These microscopic appearances, then, (if such be the nature of the disease and its progress in the kidney,) are highly important in reference to prognosis.

It remains to examine some of the secondary diseases, as they are termed, such as dropsy, hypertrophy of heart, and coma, in connection with the foregoing pathological views of their morbid condition. It has been stated that the appearance of dropsy was first observed in each of these cases, in the extremities, as the hands and feet. This circumstance would indicate that there existed a condition of the blood favorable to the transudation of its watery portions; such was the case; for the blood becomes more and more impoverished by the constant out-pouring of the albumen in the renal secretion; and besides this, the capillary circulation, generally is impeded by the presence, in the blood, of excrementitious materials, which, in their normal condition, are separated by the kidneys. The latter cause would account for that remarkable phenomenon of the disease, hypertrophy of the heart uncomplicated with dilatation or valvular lesions; the heart having to exert more force in propelling the blood through the arteries on account of the impeded capillary circulation, as first suggested by Dr. Bright.

The sudden development of serous inflammation and of coma, also, is due to the circulation in the blood of foreign materials, as urea for instance. The cerebral symptoms are most likely to supervene, in those cases where there has been a scanty secretion of urine.

Taking the same view of the nature of the disease, it would be interesting to compare the remaining symptoms with this condition of the blood; but these points, with many others, will have to be omitted in order to call attention to a few remarks on the ultimate cause, or causes, of Bright's disease. The skin and kidneys possessing, to a great degree, a vicarious action, any circumstances tending to affect injuriously the surface of the body, might give rise to renal disease; thus exposure to cold, and wet, as was the case in Nos. II and III; the exanthematous fevers, as scarlatina. Circumstances which impoverish the blood may give rise to this disease, as defective nutrition, the latter having numberless causes for its production: one of the most frequent of these causes, is the intemperate use of alcoholic drinks,as was noted in all three of the cases. There may be many other ultimate causes, as gout, rheumatism, erysipelas, &c., all of them, it would seem, tending "to produce a morbid state of the blood."

